# The trade-off between photosynthetic rate and thallus moisture-demand explains lichen habitat association with the temperate rainforest

**DOI:** 10.1007/s00442-025-05687-3

**Published:** 2025-03-05

**Authors:** Amaris Ormond, Christopher J. Ellis, Claudia Colesie

**Affiliations:** 1 School of Geosciences, Global Change Institute, University of Edinburgh, Edinburgh, UK; 2 Royal Botanic Garden Edinburgh, 20A Inverleith Row, Edinburgh, EH3 5LR UK

**Keywords:** Ecophysiology, Epiphytes, Lichens, Temperate rainforests, Water relations

## Abstract

Temperate rainforests are globally rare, covering less than 1% of the Earth’s surface, with 15% of their suitable climate space located in Europe. These ecosystems are uniquely defined by diverse cryptogamic species, particularly epiphytic lichens, which play crucial roles in forest biodiversity, trophic interactions, and biogeochemical processes. However, the ecophysiology of temperate rainforest lichens, with the potential to explain their regional to local distribution, their primary productivity, growth rates and biomass accumulation, remains under researched. This study asked whether the coexistence of morphologically different species within Scotland’s temperate rainforest is driven by adaptation and subsequent species-sorting into contrasting microclimatic moisture environments. We examined seven species, analysing their biogeographic distributions and physiological responses to controlled moisture and light gradients to understand their degree of association with the temperate rainforest habitat. Our results indicated that species with the strongest temperate rainforest association had higher thallus water requirements (OptWC, MinWC and MaxWC) to achieve maximal photosynthesis (MaxNP) and that these appeared mediated by morphological traits. We found that, when morphological traits relating to water capture and retention, including layers of rhizine and tomentum, are linked to physiological optima, they can begin to explain how species across a spectrum of morphologies are differentially adapted or acclimated and associated to different degrees with the temperate rainforest climate. These findings also underscore the significance of ecophysiological knowledge for predicting the impacts of climate change on temperate rainforest biodiversity, since species’ microhabitat responses will be pivotal in understanding broader ecological shifts.

**Supplementary Information:**

The online version contains supplementary material available at 10.1007/s00442-025-05687-3.

## Introduction

Temperate rainforest is a globally rare habitat and the specific climate conditions that support its occurrence occur across less than 1% of the Earth’s surface (DellaSala 2011). Fifteen% of the climate space suitable for development of temperate rainforest lies within Europe. Ecologically, European temperate rainforest comprises broadleaf and mixed woodland, including for example, sessile oak (*Quercus petraea*), downy birch (*Betula pubescens*), ash (*Fraxinus excelsior*), alder (*Alnus glutinosa*), hazel (*Corylus avellana*) and Scots pine (*Pinus sylvestris*). However, European temperate rainforest is uniquely characterised by an assemblage of cryptogamic species, inclusive of abundant communities of epiphytic lichens and ground-layer bryophytes. It is becoming increasingly apparent that these cryptogamic species contribute important functions to forest ecosystems (Qu et al. 2024). In the case of lichen epiphytes: (i) they contribute significantly to the local biodiversity (Affeld et al. 2008), (ii) they comprise a substantial proportion of above ground forest biomass (Díaz et al. 2010; Price and Hochachka 2001), (iii) they occupy key nodes in trophic webs and are a source of food and habitat for associated forest species, from ungulates (Thompson et al. 2015) to nematodes and gastropods (Baur and Baur 1997; Bokhorst et al. 2015), and (iv) they are acknowledged to be of general importance in global-to-local biogeochemical (Porada et al. 2014) and hydrological cycles (Porada et al. 2018; Pypker et al. 2006). Furthermore, the presence of epiphytic lichens directly alters the within and beneath-canopy microclimate through retention and evaporation of water and a shading effect that buffers temperature fluctuations (Stanton et al. 2014). In characterising the lichen epiphyte response to climate, there is evidence that species phenotypes are divergently adapted to contrasting climates and microclimates (Gaio-Oliveira et al. 2004; Stanton et al. 2023). This makes certain phenotypic traits potentially useful for developing a functional climatic response that can improve upon the correlative distributions behind bioclimatic models. Developing a functional understanding of species response may be especially important if the future climate within the European temperate rainforest zone has no clear present-day analogue (Ellis and Eaton 2018), rendering extrapolation of correlative distributional patterns especially problematic. Accordingly, this study carried out a series of experiments aiming to improve the functional understanding of lichen epiphyte association with European temperate rainforest habitat, based on contrasts in distribution for species with a range of phenotypes, and with application at the microhabitat scale.

Arguably the most relevant climatic factor influencing lichen epiphyte ecophysiology is the moisture regime of the environment (Hovind et al. 2020). Lichens are poikilohydric, and they equilibrate their water status with the surrounding environment. Wet conditions can lead to physiological activity, followed by periods of oversaturation limiting photosynthesis (Lange et al. 2001), whilst dry conditions lead to desiccation accompanied by physiological dormancy (Cowan et al. 1992, Lange et al. 1998, Lange et al. 1999). Thallus rehydration has potential to produce a strong resaturation respiration peak, which is amplified by elevated daytime and nocturnal temperatures (Bidussi et al. 2013). The hydration status of a lichen and its subsequent photosynthetic activity is also determined importantly by the photobiont. Cyanolichens need water in liquid form for photosynthetic reactivation whilst chlorolichens can reactivate from water vapour alone (Lange 1980, Lange et al. 1998, Lange et al. 2001).

Light is another factor driving lichen epiphyte photosynthesis. Light saturation curves (LSC) in lichens are analogous to those of vascular plants, displaying characteristic traits of sun and shade adaptation (Green et al. 1997; Osyczka and Myśliwa-Kurdziel 2023). For New Zealand’s evergreen temperate rainforest lichens for example, those species that inhabit shaded microclimates tended to have lower light compensation and saturation points than species inhabiting open, light exposed areas of the forest (Green et al. 1997). Lichen species appear to have adaptive or acclimatory phenotypic traits (Gauslaa and Coxson 2011) that optimise hydration within a given moisture environment (Lange et al. 1999). For example, a coupled moisture-light gradient can cause acclimation in lichen morphology. Lichens experiencing high irradiance (and consequently drier) microclimates respond by increasing thallus thickness and subsequently thallus mass to area ratio to increase water capture and storage (Green and Lange 1991; Hilmo 2002) and species such as *Lobaria pulmonaria* are documented to also increase synthesis of protective pigments to reduce photoinhibitionary effects (Gauslaa and Solhaug 2004). Ultimately, lichen growth and reproduction are dependent on the rate of carbon acquisition, which is a balance between carbon gain from net photosynthesis (NP) and loss related respiration (DR), both of which are intrinsically linked to local microclimatic conditions. NP and carbon gain will be maximised when both thallus water status and irradiance are optimal, with lichens achieving this optimisation for a given moisture environment through phenotypic (morphological and ecophysiological) adaptation and acclimation.

The temperate rainforest presents a special opportunity to interrogate the role of phenotypic adaptation and acclimation in driving the lichen epiphyte response, and particularly the importance of morphology, ecophysiology, and their interdependency. This is because the temperate rainforest epiphyte assemblage includes lichen species that are very different in their overall morphologies and in their degree of association with the temperate rainforest climate. The temperate rainforest assemblage includes thin highly branched hair-lichens such as *Usnea* species (e.g. *Usnea flammea*) through to species with a very high Water Holding Capacity (WHC) such as *Leptogium* species (e.g. *Leptogium burgessii*) (Ellis 2016). These same species include a spectrum of anatomical features that have been cited as adaptations to varying moisture environments, including the occurrence of tomentum (Gauslaa and Coxson 2011), rhizines (Gauslaa and Solhaug 1998) or a hypothallus (Benson and Coxson 2002). We propose that these features explain whether species are strongly or only weakly associated with the temperate rainforest, and that they will also influence species microhabitat distribution within the rainforest climatic zone.

To better understand the role of phenotypic adaptation and acclimation in characterising the lichen epiphyte microclimatic response, we: 1. identified seven morphologically contrasting temperate rainforest species that showed varying degrees of habitat association, and that could be considered along a spectrum from ‘obligate’ to ‘facultative’ temperate rainforest species, 2. we tested whether these species could be similarly ordered with respect to a set of core parameters that indexed phenotypic measurements in their morphology and ecophysiology. Our hypothesis was that despite being representative components of the temperate rainforest assemblage, the selected species would be significantly different in these core parameters. This would be consistent with adaptation and species-sorting to contrasting microclimatic moisture environments, explaining the coexistence of phenotypically different species within the temperate rainforest assemblage.

## Materials and methods

### Species selection and sampling

**Fig. 1:**
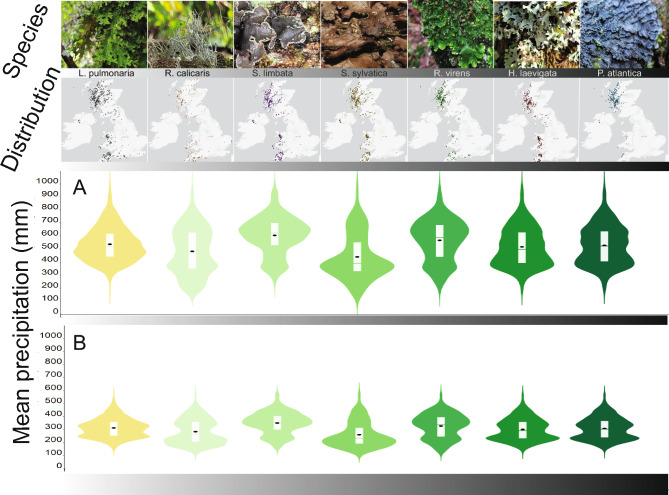
Species distribution patterns indicate subtle shifts in the precipitation regime occupied. In situ photographs of the studied species ranked left to right by increasing habitat association to the (higher moisture) temperate rainforest climate (upper row) and their UK distribution maps (second row; GBIF data). Violin plots show the occurrence data linked with CHELSA bioclimatic layers for precipitation of the wettest quarter of the year (**A**) and precipitation of the warmest quarter of the year (**B**)

Seven case-study species were selected (Fig. 1) based on their statistical association with a standard model of the temperate rainforest climate (Ellis 2016). The species had different strengths of habitat association with temperate rainforest spanning from highly restricted to a hyper-oceanic zone, to lesser restricted species occurring beyond the temperate rainforest zone in scattered but locally suitable habitats. We tested and visualised this strength of association using British climate space and in rank-order of their habitat association with the temperate rainforest from low to high, the selected species were:

- *Lobaria pulmonaria* (L.) Hoffm., a foliose cephalolichen with a primary trebouxoid photobiont and cyanobacteria within internal cephalodia (Brodo 2016; Jordan 1973). *L. pulmonaria* tends to be loosely attached to its bark substratum as an epiphyte, and features a prominent reticulum of deep depressions and ridges across the thallus surface (Phinney 2019).
- *Ramalina calicaris* (L.) Röhl / (L.) Fr., a fruticose chlorolichen with a trebouxoid photobiont. It grows upright or pendant and is characterised by deep, longitudinal channels along the branches and frequent marginal apothecia and occasional pseudocyphellae (Oh et al. 2014).
- *Sticta limbata* (Sm.) Ach., a foliose cyanolichen with *Nostoc,* cyphellae on the underside of the thallus, and with a fine layer of tomentum (Galloway 1994).
- *Sticta sylvatica* (Huds.) Ach., a foliose cyanolichen with *Nostoc.* The lower surface features tomentum with dispersed cyphellae (Galloway 1994).
- *Ricasolia virens* (With.) H.H.Blom & Tønsberg, a foliose cephalolichen with a primary trebouxoid photobiont and cyanobacteria *Nostoc* within cephalodia (Moncada et al. 2013), it has a prostrate thallus which is often tightly adhered to its bark substratum and having frequent apothecia (Longinotti et al. 2017).
- *Hypotrachyna laevigata* (Sm.) Hale, a foliose chlorolichen with a green algal trebouxoid photobiont and dichotomously branched rhizines on the under surface (Jayala et al 2013; Ahmadjian 1993).
- *Pectenia atlantica* (Degel.) P.M.Jørg., L.Lindblom, Wedin & S.Ekman, a foliose cyanolichen with *Nostoc* (Cannon et al. 2021). It is an isidiate species with a thick water-holding rhizine layer referred to as a hypothallus (Gauslaa and Solhaug 1998).

**Fig. 2:**
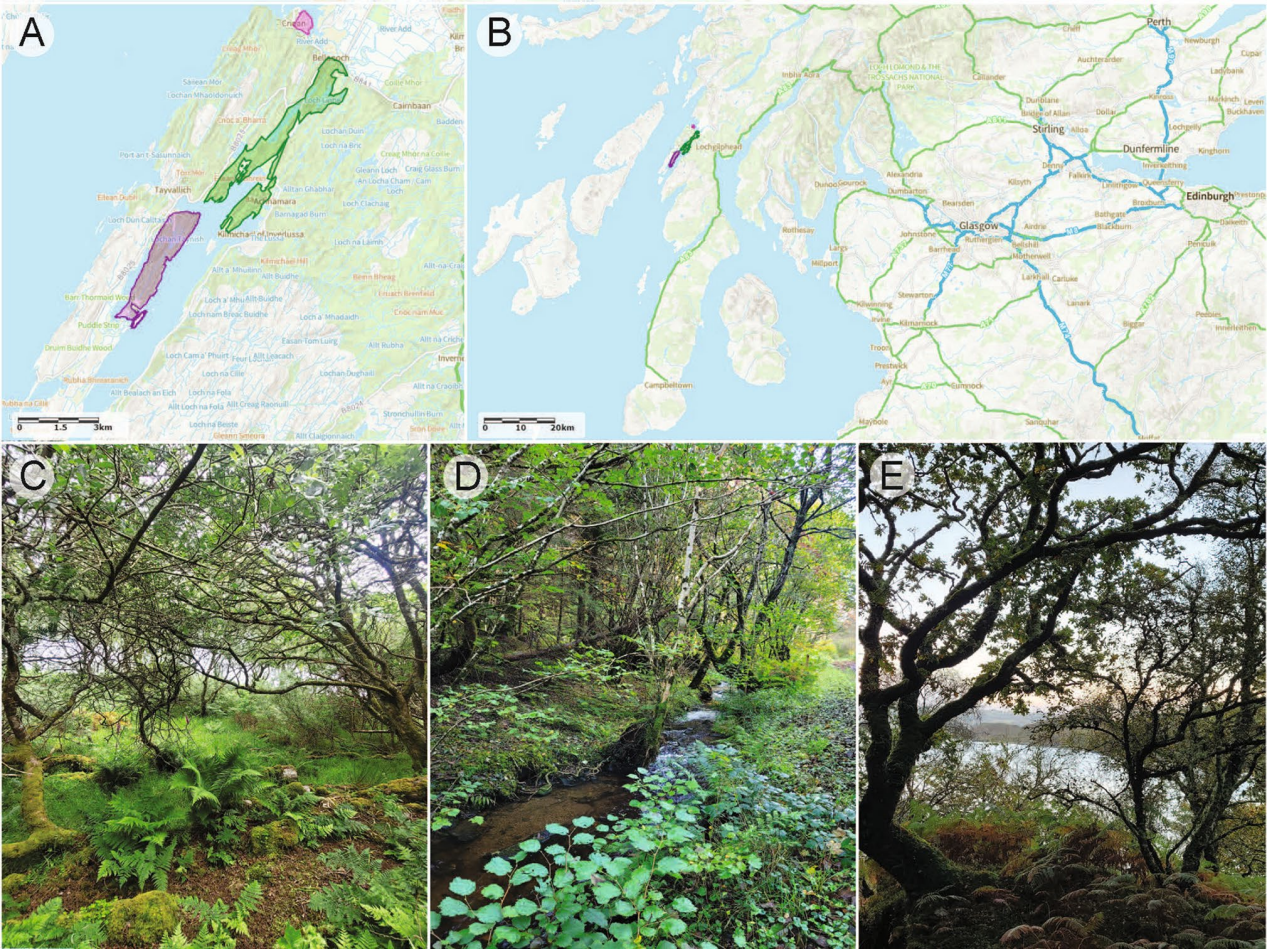
The temperate rainforest habitat. **A** Sites for collection of experimental specimens were located along the Scottish West Coast within Argyll and Bute, Scotland, United Kingdom. Taynish (purple), Knapdale (green) and Crinan (pink). **B** The location in the wider context within Scotland. Maps created using the Multi-Agency Geographic Information for the Countryside. Natural England. **C** shows the study site at Taynish, **D** Knapdale and **E** Crinan

Between the 8^th^ and 10th October 2021, one sample of each species was collected from each of the three temperate rainforest sites situated in the district of Argyll and Bute, Western Scotland, United Kingdom (Fig. 2, Taynish National Nature Reserve (NNR) (56.007°N, 5.631°W), Knapdale Woods SSSI (56.05670°N, 5.56150°W), and Crinan Wood (56.08668°N, 5.55428°W)). The collection sites are internationally important for their temperate rainforest lichen epiphyte communities and incorporated within the UK conservation network {Coppins, 2006 #193} (Coppins and Coppins 2005; Ellis et al. 2015). Three (*n* = *3*) thalli of each species were collected before being air dried and freezer stored (− 20 °C) until they were analysed. The maximum storage period was no longer than 3 months.

### Quantifying the species’ realised niche

To quantify each species’ realised niche, distribution patterns were mapped using occurrence data obtained from the Global Biodiversity Information Facility (GBIF, 2021). The British distributions were sub-sampled for the period 1979–2013 for all seven species. Data were cleaned to remove erroneous (non-terrestrial coordinates) or duplicates and then mapped in ArcGIS 3.0.1 (ESRI, 2022). Considering covariation across multiple climate variables, each species’ realised niche within Scotland (the regional study area) was summarised by comparing species occurrence with precipitation data from CHELSA; specifically, bio16 (precipitation of the wettest quarter) and bio18 (precipitation of the warmest quarter) (Karger et al. 2017, 2018). These two datasets were chosen because they represent two distinct seasons in which lichens could be differently affected by changes in the moisture environment (Ellis 2019).

### Quantifying the species’ fundamental niche

To describe each species’ fundamental niche, we measured the water relations, light economics and carbon acquisition as three different metrics being pivotal to a species’ ecophysiology (Table 1). The measurements were done under controlled laboratory conditions using a minicuvette system (GFS 3000, Walz GmbH, Effeltrich, Germany). From the measurements we then extracted a set of core parameters to describe key features in the lichen species response. Prior to the gas exchange measurements, samples were removed from freezer storage, placed into refrigeration storage (5 °C), at low light (25-µmol photons m^−2^ s^−1^) for 48 h. They were then rewetted with water collected from a small waterfall in the Taynish field site and stored in the fridge for a further 24 h. Samples were kept refrigerated between measurements.

**Table 1:** Lichen measurements categorised into three ecophysiological metrics: water economics, light economics and carbon acquisition

Function	Core parameters	Unit of measurement	Physiological function
Water relations	Maximum water content (MaxWC)	mm H_2_O precipitation equivalent	Upper level of water supply for 90% MaxNP
	Minimum water content (MinWC)	mm H_2_O precipitation equivalent	Lower level of water supply for 90% MaxNP
	Range of optimal water content (OptWC)	mm H_2_O precipitation equivalent	Width of water response amplitude
Light economics	Light compensation point (LCP)	μmol photons m^−2^ s^−1^ PAR	Low light use efficiency
	Light saturation point (LSP)	μmol photons m^−2^ s^−1^ PAR	Light use
Carbon acquisition	Maximum net photosynthesis (MaxNP)	nmol g^−2^DM s^−1^	Maximum carbon gain
	Dark respiration (DR)	nmol g^−2^DM s^−1^	Maintenance and growth
	Carbon gain efficiency (CGE)	MaxNP / DR	Overall carbon gain
	Chlorophyll-content (Chl)	μg Chl/ mg DM^−2^	Increase carbon gain / indicator of photosynthetic capacity

Units of measurement and their function for lichen growth and fitness are shown

Measurements started by wetting individual samples and then recording the mass (mg) of a gently shaken but fully hydrated sample (including internal and external water). Each sample was then individually placed into the cuvette and a series of light response curves were measured until the sample was dry and dormant, producing a series of response curves across a range of different levels of thallus hydration. The light curves were conducted with steps of increasing photosynthetic photon flux density (PPFD; 0, 12, 25, 50, 100, 300, 500, 1000, 1500-μmol photons m − 2 s − 1) at a temperature of 18 °C, 90% relative humidity, and ambient CO_2_. Samples were exposed to each PPFD step until the lichen assimilation rates had stabilised. Between each light response curve, the sample was weighed (mg) and these mass measurements were later used to calculate the water content (WC) of the sample during each light curve.

Measurements were completed for three (*n* = 3) replicates of each species, except for *R. virens* (*n* = 2). Following the gas exchange measurements, each fully hydrated thallus was photographed and the surface area was measured in cm^2^ using the software ImageJ (Fiji) 2.5. Once these measurements were completed, the samples were placed in a drying oven at 60 °C for 48 h, and the dry mass (DM) (mg) was determined (Online material 1).

### Core parameters

Net photosynthesis (NP) and dark respiration (DR) were expressed per unit of dry mass (DM) and per unit area (Online material 2). Core parameters for photosynthesis were determined from measurements at optimal WC and under saturating light conditions. Therefore, first, the light saturation points (LSP), the light intensity at which 90% of maximum NP was reached, was calculated. Once the saturated light had been established, the optimal water content (OptWC) was determined. This was the range of WC between MinWC (the lower level of WC resulting in NP greater than 90% of MaxNP) and MaxWC (the upper level of WC resulting in 90% of NPMax). The thallus WC in mm rainfall equivalent was calculated for each of these core parameters based on the thallus area and mass (Gauslaa 2014; Gauslaa et al. 2017). From this dataset, we also extracted the light compensation points (LCPs), the light intensity at which NP becomes positive. Finally, carbon gain efficiency (CGE) was calculated as NP/DR ratio.

The sample chlorophyll contents were determined by extracting the entire thallus twice with dimethyl sulfoxide (DMSO) and magnesium carbonate (MgCO_3_) as a buffering agent; at 60 °C for 60 min. Absorption was then measured at standard wavelengths (Ronen and Galun 1984) and chlorophyll content (Chl-a + b / Chl-a) was calculated (Arnon and McSwain 1974). Morphological parameters of Specific Thallus Mass (STM) and (WHC) were calculated from thallus mass and area measurements and WHC is presented as mm rainfall equivalent for context.

### Statistical data analysis

Data for all core parameters (LSP, LCP, MinWC, MaxWC, OptWC, MaxNP, DR, CGE and chlorophyll content) were summarised as the statistical mean ± 1SE. The data were then tested for normality (Shapiro–Wilk) and appropriate data transformations applied where normality was not obtained. *R. virens* was not tested for normality because the sample size (*n* = 2) was too small. A one-way Analysis of Variance (ANOVA) was performed to test differences in mean values of core parameters at a significance level of p < 0.05, aiming to identify any significant differences amongst the seven species. Post-hoc Tukey tests were performed where significance was observed.

To test if there was a relationship between the strength of a species’ habitat association with the temperate rainforest climate, and each of the core parameters. Species were ranked from low to high in their degree of association to the temperate rainforest and an ordered logistic regression was performed using the ‘*polr*’ function of the ‘*MASS*’ package in R. All tests were analysed using a significance level of *p* < 0.05 and figures were produced using the ‘ggplot2’ package in R (Wickham 2016). The data and code to reproduce the data analysis can be found on GitHub: https://github.com/AmarisOrmond.

## Results

### Realised niche

We confirmed that all selected study species occur in the western and oceanic zone of Britain (Fig. 1) consistent with their status as components of the temperate rainforest lichen epiphyte assemblage. However, as expected, there were also differences in their overall distributions, with some species being more widespread and occupying a broader realised niche space. For example, the distribution of the cyanolichen *P. atlantica* is apparently more restricted than that of *S. limbata*, whilst the distribution of the cephalolichen *L. pulmonaria* is less restricted than that of *R. virens*. The distribution of species is characterised by a precipitation range that becomes narrower for the species most strongly associated with the temperate rainforest habitat (Fig. 1A and B).

### Fundamental niche

**Fig. 3:**
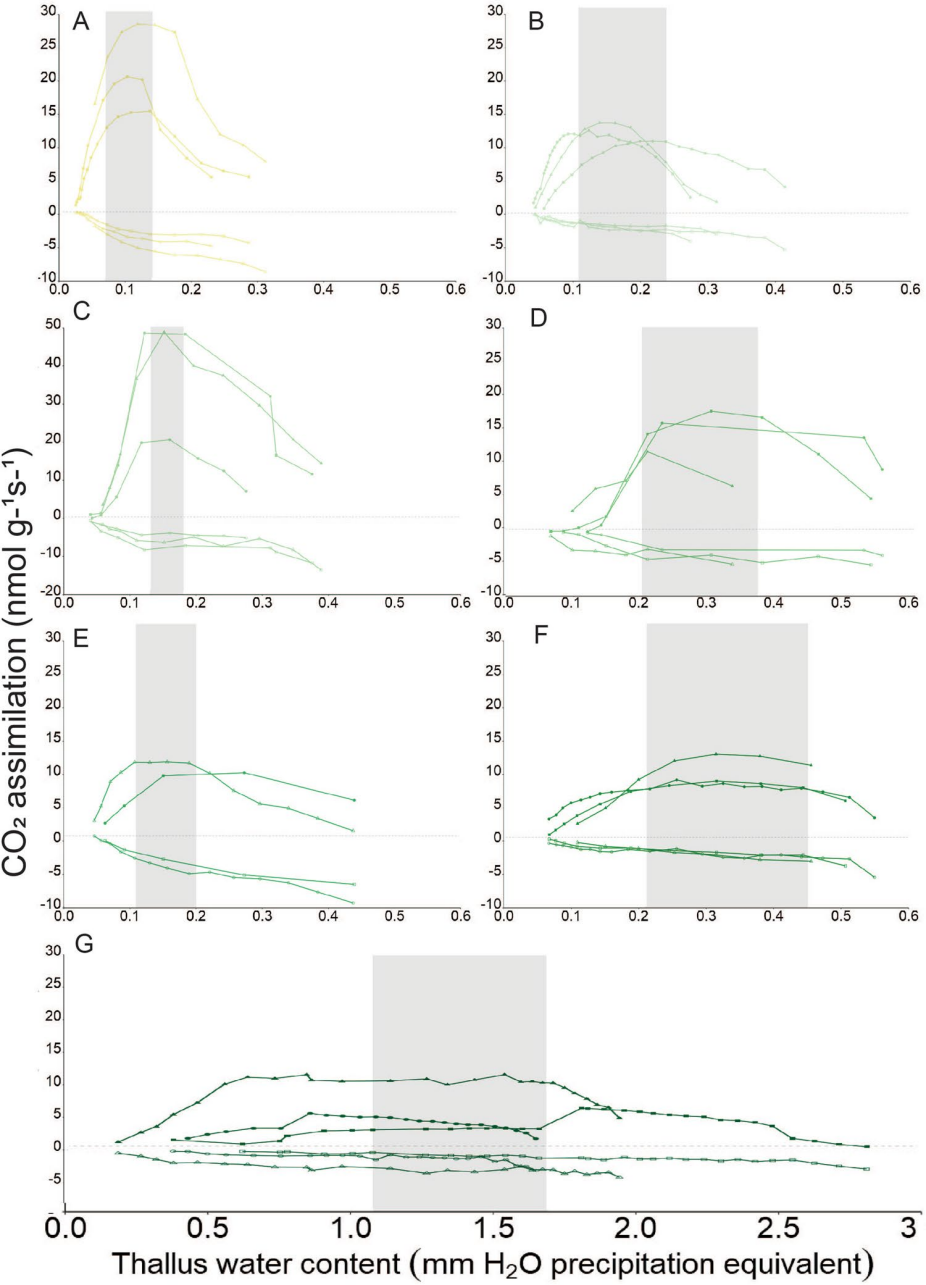
Water economics are species specific. The responses of net photosynthesis (positive CO_2_ assimilation) and dark respiration (negative CO_2_ assimilation) to changes in thallus water content for (**A**) *L. pulmonaria*, (**B**) *R. calicaris*, (**C**) *S. limbata* with a larger scale for CO_2_ assimilation, (**D**) *S. sylvatica,* (**E**) *R. virens*, (**F**) *H. laevigata* and (**G**) *P. atlantica* (different scale). Each line colour represents one individual sample. The optimal thallus water content range based on a mean average of all three samples (*n* = 3) is indicated in grey

**Fig. 4:**
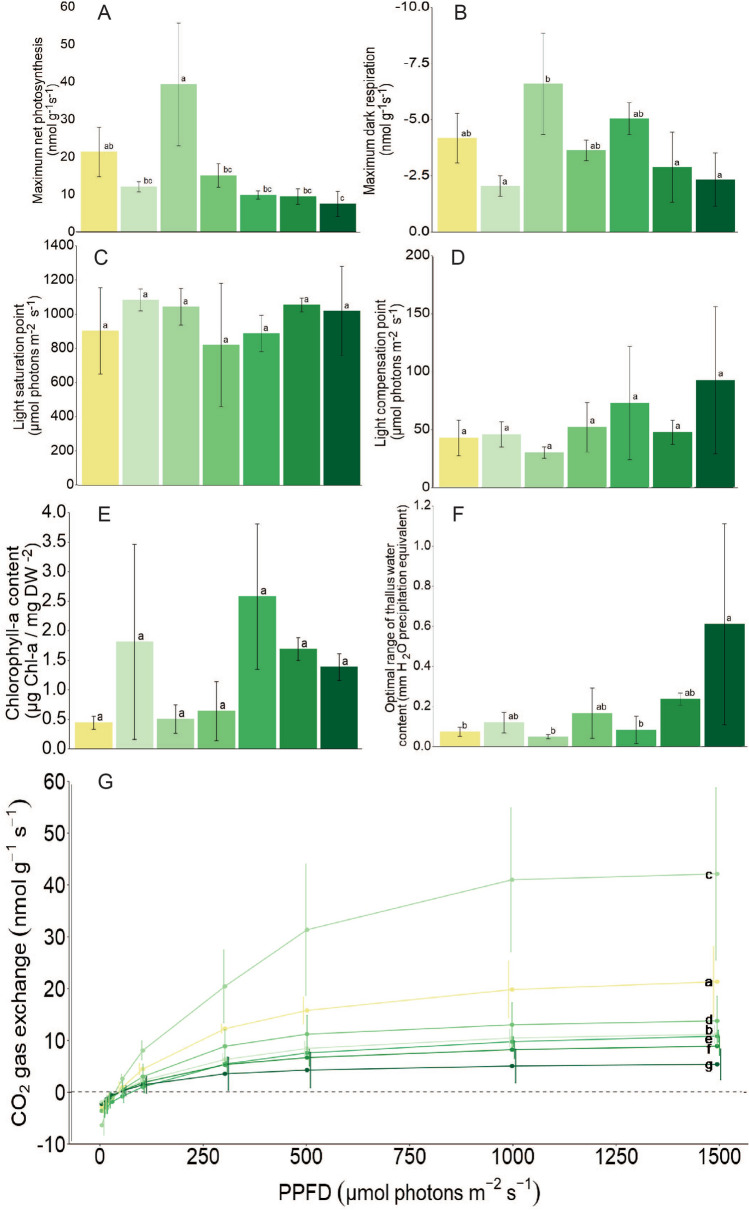
Core parameters of CO_2_ gas exchange for the seven study species ranked left to right in each graph by increasing habitat association to the temperate rainforest climate (*L. pulmonaria*, *R. calicaris*, *S. limbata*, *S. sylvatica*, *R. virens*, *H. laevigata*, *P. atlantica*). (**A**) maximum net photosynthesis, (**B**) maximum dark respiration, (**C**) the light saturation point and (**D**) the light compensation point, chlorophyll-a content (**E**) and the optimal range of thallus water content (**F**). All data represent mean ± SD values for *n* = 3 (except *R. virens* where *n* = 2) and statistically significant differences are indicated by letters, values with the same letters are not significantly different. (**G**) The physiological response of CO_2_ gas exchange to stepwise increase of photosynthetic flux densities (PPFD, μmol photons m^−2^ s.^−1^) measured at optimal thallus water contents. Species are indicated using letters (*a-g*)

Net photosynthesis of all species measured at 18 °C and saturating PPFD responded to thallus WC with typical saturation curves (Fig. 3). A decrease in NP at high (suprasaturated) and low (desiccated) water contents, and a peak in NP around an optimum WC is evident in all species. DR also follows a typical saturation curve for all species, increasing with increasing thallus WC, up until it reaches a maximal rate, from which it remains relatively constant despite further increases in hydration. MaxWC and MinWC were significantly higher (F = 22.73, *p* =  < 0.001 and F = 30.14, *p* =  < 0.001) for *P. atlantica* (1.69 / 1.08 mm H_2_O equivalent) when compared to all other species. Minimum thallus WC when measured as a per cent (MinWC%) was significantly higher for *P. atlantica* than all other species (F = 27.15, *p* =  < 0.001), whereas MaxWC% was significantly lower for *L. pulmonaria* (125%) and *R. calicaris* (117%), when compared to *S. sylvatica* (280%), *H. laevigata* (275%) and *P. atlantica* (487%). *P. atlantica* therefore required more moisture to achieve MaxNP than all other species and its minimum water requirement for MaxNP was four times larger than that of any other species (1.08 mm / 280%). *L. pulmonaria*’s MinWC was lower than all other species (0.07 mm / 59%) and significantly lower than for *S. sylvatica* (0.20 mm / 153%) and *H. laevigata* (0.21 mm / 127%). *R. calicaris* had the second lowest MinWC at 0.11 mm / 66%. OptWC (maximum—minimum WC) was significantly broader (F = 5.108, *p* =  < 0.001) for *P. atlantica* (0.62 mm) than for *L. pulmonaria* (0.08 mm), *S. limbata* (0.05 mm) and *R. virens* (0.08 mm), but not significantly broader than for *H. laevigata* (0.24 mm), *S. sylvatica* (0.17 mm) or *R. calicaris* (0.12 mm) (Table 2 and Fig. 4F). When considering OptWC as a % of thallus DM (OptWC%) there was no significant relationship (F = 1.075, *p* = 0.426), although it tended to increase with strength of temperate rainforest association, from *R. calicaris* at 51% to *P. atlantica* at 207%, with the least-associated species *L. pulmonaria* being an exception to this with an OptWC% of 65%.

**Table 2:** Summary of moisture related core parameters of photosynthesis, Specific Thallus Mass (STM) and Water Holding Capacity (WHC) for all study species

Species	MaxWC (mm) and (%)	MinWC (mm) and (%)	OptWC (mm) and (%)	STM	WHC (mm H*2*O per cm^2^)	MaxNP (nmol g − ^2^DM s^−1^)	CGE
*Lobaria pulmonaria*	0.14 ± 0.03^a^ 125 ± 18.0^a^	0.07 ± 0.00^a^ 59 ± 1.5^a^	0.08 ± 0.02^b^ 65.6 ± 16.7^a^	11.4 ± 0.8^ab^	0.31 ± 0.0^a^	21.2 ± 6.6^ab^	5.1 ± 0.5^ab^
*Ramalina* *calicaris*	0.24 ± 0.07^abc^ 117 ± 15.0^a^	0.11 ± 0.03^abc^ 66 ± 15.5^a^	0.12 ± 0.05^ab^ 51 ± 26^a^	17.2 ± 1.6^b^	0.36 ± 0.1^ab^	11.9 ± 1.3^bc^	6.2 ± 2.0^a^
*Sticta* *limbata*	0.18 ± 0.02^ab^ 225 ± 102.9^ab^	0.13 ± 0.02^ab^ 162 ± 20.6^b^	0.05 ± 0.01^b^ 63 ± 13.9^a^	7.9 ± 0.1^a^	0.38 ± 0.1^ab^	39.1 ± 16.3^a^	5.9 ± 1.3^ab^
*Sticta sylvatica*	0.37 ± 0.16^bc^ 280 ± 86.0^bc^	0.20 ± 0.03^bc^ 153 ± 24.4^b^	0.17 ± 0.13^ab^ 127 ± 100.1	13.9 ± 1.0^b^	0.63 ± 0.1^c^	14.9 ± 3.1^bc^	4.1 ± 1.1^ab^
*Ricasolia virens*	0.20 ± 0.04^abc^ 148 ± 14.9^ab^	0.11 ± 0.03^abc^ 70 ± 9.2^a^	0.08 ± 0.07^b^ 78 ± 24.0^a^	16.9 ± 4.2^b^	0.52 ± 0.1^abc^	9.8 ± 1.1^bc^	2.0 ± 0.5^b^
*Hypotrachyna laevigata*	0.45 ± 0.00^c^ 275 ± 61.5^bc^	0.21 ± 0.04^c^ 127 ± 26.2^b^	0.24 ± 0.03^ab^ 148 ± 36.0^a^	16.5 ± 2.7^b^	0.57 ± 0.0^bc^	9.4 ± 2.0^bc^	3.9 ± 1.8^ab^
*Pectenia atlantica*	1.69 ± 0.47^d^ 487 ± 158.2^c^	1.08 ± 0.7^d^ 280 ± 76.6^c^	0.62 ± 0.5^a^ 207 ± 215.4^a^	36.2 ± 12.3^c^	2.19 ± 0.6^d^	7.4 ± 3.3^c^	3.3 ± 0.2^ab^

The water content (WC) is calculated as a function of thallus dry mass (mg DM) and represented as mm precipitation equivalent (mm H2O) and % of thallus DM. Data represent the means of (*n* = 3) (*R. virens* *n* = 2) ± standard deviation. Values with the same small letter are not significantly different. Species are ordered based on increasing habitat association with the temperate rainforest, from weakly (top) to strongly associated (bottom)

Response of CO_2_ gas exchange to increasing PPFD showed a typical saturation curve for all seven species when measured at 18 °C and optimum WC (Fig. 4G). There was no evidence of photoinhibition even at the highest PPFD (1500-μmol photons m^−2^ s^−1^ PPFD). The LCP was similar for all species and showed no significance differences between species (*F* = 1.338*, p* = 0.309, Fig. 4D). The light saturation point (LSP) was also similar between species (*F* = 0.67*, p* = 0.674). LCP is reached > 30 PPFD for all species and in general LSP was high across all species and ranged from 825 – 1090 μmol photons m^−2^ s^−1^ PPFD (Fig. 4C).

Mean maximum rates of net photosynthesis (MaxNP) (nmol g^−2^DM s^−1^) were significantly different between species (*F* = 9.373*, p* =  < 0.001) (Fig. 4A and Table 2). MaxNP was highest for *L. pulmonaria, S. limbata* and *S. sylvatica* (21.2, 39.1 and 14.9 nmol g^−2^ DM s^−1^) and lowest for *H. laevigata* and *P. atlantica* (9.4 and 7.4 nmol g^−2^ DM s^−1^). The MaxNP of *S. limbata* (39.1 nmol g^−2^ DM s^−1^) was significantly greater than all other species apart from *L. pulmonaria.* Maximum rates of dark respiration (DR) varied significantly between species (*F* = 4.532*, p* = 0.010, Fig. 4B). *Sticta limbata* had the highest rates of DR (−6.5 nmol g^−2^ DM s^−1^), with these being significantly higher than for *R. calicaris* (−2.0), *H. laevigata* (−2.9) and *P. atlantica* (−2.9). Chlorophyll per sample DM (μg Chl-a + b/mg DM^−2^) / (μg Chl-a/ mg DM^−2^) did not vary significantly between species (F = 1.859, *p* = 0.164) / (F = 2.737*, p* = 0.060, Fig. 4E). Carbon gain efficiency (CGE) was significantly different between species (*F* = 3.398*, p* = 0.030) and was highest for *R. calicaris* (6.2), which was in turn significantly higher than for *R. virens*. Morphological parameters of STM varied significantly between species (F = 9.452, *p* =  < 0.000). *P. atlantica* had most biomass per unit area (36.2 mg cm^2^), whilst *S. limbata* had significantly lower STM than other species except *L. pulmonaria.* WHC significantly varied between species (F = 37.3 *p* =  < 0.000) and was greatest for *P. atlantica* (2.2 mm H_2_O per cm^2^) and lowest for *L. pulmonaria* (0.3 mm H2O per cm^2^). The WHC of *P. atlantica* was higher than that of all other species, whereas *S. sylvatica* had a greater WHC (0.6 mm H2O per cm^2^) than *L. pulmonaria, R. calicaris* and *S. limbata.*

### Habitat association analysis

Tested using ordinal regression the most strongly significant positive relationships between the species’ habitat association with temperate rainforest, and core parameters, was for parameters that represented water relation values: MaxWC (coeff = 12.87, *t* = 2.92, *p* = 0.004), MinWC (coeff = 36.17, *t* = 3.13, *p* = 0.002) and OptWC (coeff = 16.84, *t* = 2.97, *p* = 0.003). There was a weaker positive relationship with LCP (coeff = 0.031, *t* = 2.036, *p* = 0.042) and a significant negative relationship between the species’ habitat association with temperate rainforest and CGE (coeff = −0.587, *t* = −2.411, *p* = 0.002). Other core parameters were non-significant in this regard: LSP (*p* = 0.816), MaxNP (*p* = 0.084), DR (*p* = 0.34), and Chl (*p* = 0.117).

## Discussion

This study investigated how the ecophysiology of lichen epiphytes may be adapted to the temperate rainforest climate, though for species different degrees of habitat association that could also explain their divergent adaptation to local microclimates. We were able to identify differences in the biogeographic distributions of seven study species that are suggestive of a subtly contrasting adaptation to climatic wetness, and thereby confirming a useful test case in which the species could be ranked according to their strength of habitat association with the temperate rainforest climate. Core parameters which we propose as reflecting ecophysiological adaptation and/or acclimation, were extracted from controlled measurements of each species’ response to moisture and light gradients for a given temperature.

It is important that we observed a well-supported lichen response to moisture and light gradients, validating the experimental outcomes. This included a characteristic response of net photosynthesis (NP) and dark respiration (DR) to thallus WC from liquid water, with NP increasing with WC, before a clear depression of NP at high thallus WCs, likely associated with suprasaturation and diffusion resistance of CO_2_ to the photobiont (Lange et al. 2001, 2004; Pintado and Sancho 2002). Coincidingly, upon initial hydration of the thalli, an increase in DR, likely attributed to non-metabolic release of CO_2_ and subsequent resaturation–respiration was observed, a phenomenon common to lichens, particularly under laboratory conditions (Lange 2002, Scheidegger et al. 1995; Sundberg et al. 1999). Comparable patterns are reported across lichens sampled from many different habitats, encompassing species with varying morphologies, within both laboratory and natural settings, inclusive of temperate (Lange et al. 1993) and tropical rainforests (Lange et al. 2004), as well as contrasting, drier habitats (Büdel et al. 2013; Lange et al. 2006; Schipperges 1992). Similarly, the species light responses all displayed a characteristic increase in NP with increasing PPFD up to a saturation point, where NP stabilised, a pattern which is analogous to comparable studies on lichens including within rainforests (Green et al. 1997; Lange et al. 2004) and across other habitats (Lange et al. 1998, Lange and Green 1996; Tamm et al. 2018). However, when these characteristic responses were summarised for individual species as their core parameters and compared to the strength of temperate rainforest habitat association, several important observations emerge.

First, values for LCP and LSP were not significantly different amongst the species and did not therefore adequately explain species temperate rainforest habitat association, being either weakly (LCP) or not significant (LSP). These core parameters were however dissimilar to and apparently higher than those reported for different species in contrasting habitats (Gauslaa et al. 2012, Lange et al. 1994, Lange et al. 1998, Solhaug et al. 2014), and for species within disjunct tropical and (Lange et al. 2004) temperate rainforests (Green et al. 1991, 1997). One consideration for the higher LSP and LCPs reported here, comparative to rainforests in other localities, is that our sampling was from a deciduous woodland during Autumn so that study species experienced seasonal leaf abscission and an associated increase in the light environment (prior to winter darkening), which has been evidenced to increase LCP and LSPs for lichens elsewhere (Bradshaw 1965, Kershaw 1985, Lange, 2002, MacKenzie et al. 2001, Valladares et al. 2007). This aligns with knowledge that species light response could be locally adaptive or occur in acclimation to habitat-specific conditions.

Similarly, chlorophyll content (Chla) did not differ amongst species, and whilst maximal rates of DR were significantly different amongst species, the values did not explain species temperate rainforest habitat association. However, second, and in contrast to core parameters LCP, LSP, DR and Chl, the species association with temperate rainforest was significantly explained by CGE which appeared correlated (though not for *R. calicaris*) with high MaxNP (*r* = 0.525, *p* = 0.017 with 18 df), rather than with low rates of DR (*r* = 0.037, *p* = 0.878 with 18 df), as previously observed in other lichen species (Colesie et al. 2018). Furthermore, despite high rates of NP having been previously associated with high thallus chlorophyll content (Green et al. 1997; Palmqvist et al. 2002), similar to Lange et al. (2004), we observed no discernible correlation between the MaxNP and Chl_-a_ (*r* = 0.335, *p* = 0.149 with 18 df), suggesting this relationship is possibly conditional on other factors (Beckett et al. 2021).

Third, we found significant differences amongst species, and the most strongly significant effect of core parameters that characterise thallus water relations (MaxWC, MinWC and OptWC) when explaining the temperate rainforest habitat. The degree of association therefore increased for species with a higher water demand (mm equivalent) necessary to service MaxWC and MinWC. Notably, the volumes of water (MinWC and MaxWC) required for maximal photosynthesis (MaxNP) observed here, align with observations for species occurring in other habitats across known moisture gradients. Although these patterns are not exclusive, for purposes of comparison, the species most strongly associated with temperate rainforest had optimal thallus WC values (MinWC and MaxWC) comparable to chionophilous species (Schipperges 1992) as well as species from other tropical and (Lange et al. 2004) temperate rainforests (Lange et al. 1993). Similarly, the lesser associated species had MinWC and MaxWC more similar to species from drier habitats (Lange et al. 1994; Lange and Tenhunen 1982). Consistent with our results, *L. pulmonaria* has been reported previously as having a low thallus water storage capacity (Solhaug et al. 2021) and a correspondingly low MinWC for NP (Gauslaa et al. 2017). However, studies from non-rainforests report OptWC for *L. pulmonaria* of between 30 and 60% (Mafole et al. 2019; Scheidegger et al. 1995), the upper end of which is equivalent to the MinWC recorded for *L. pulmonaria* here, and confirming that this and other species are can be divergently acclimated to the moisture status of their habitat (Barták et al. 2006). This extends the consideration of MinWC and MaxWC, to OptWC, suggesting that whilst species such as *S. limbata* have high MaxNP, they do not maintain this for extended periods (low OptWC) and so have relatively short active/growth phases (Eaton and Ellis 2012), being similar in this regard to patterns reported for soil crust lichens (Lange et al. 1997; Pintado et al. 2005). When considering OptWC and WHC, both here and from other studies, the possibility of short active periods is supported by the fact that OptWC was substantially less than reported values of WHC for the same species (Gauslaa 2014; Lange et al. 2001; Solhaug et al. 2021). This suggests that these species regularly experience optimal thallus hydration at moisture levels that are below their maximal water storage (i.e. when WHC is measured for internal and external water), likely corresponding to shorter active periods in a high rainfall environment. Conversely, it highlights that species may not rely on maximising their total water storage for optimum photosynthesis and so would benefit from frequent but less intense hydration events. It is an interesting possibility that the core parameters defining thallus water relations may interact with MaxNP. For example, the species with highest MaxWC, including *P. atlantica*, although tending to have a lower rate of MaxNP, may maintain these rates over a broader range of thallus WCs (broader OptWC), thus prolonging the active period, a pattern that has been previously evidenced for *Pectenia* species (Gauslaa and Solhaug 1998).

In conclusion, our results highlight that a gradient of habitat association with the temperate rainforest, can be explained by species morphological diversity which likely enables individual species to function under the hydration regime of the temperate rainforest by sorting into contrasting microclimates. This is based on analysis of both fruticose and foliose forms with either green algal or cyanobacterial photobionts. These same species also exhibited various traits which influence water retention and capture, including tomentum, rhizines and hypothalli as well as structures such as cyphellae or pseudocyphellae, which enhance gas exchange under saturating moisture conditions (Galloway 1994; Gauslaa and Coxson 2011; Green et al. 1981). Previous studies characterising the lichen epiphyte niche in relation to functional traits have largely focussed on morphological features such as growth form, photobiont type, STM and WHC (Gauslaa and Coxson 2011; Gauslaa et al. 2017; Longinotti et al. 2017; Trobajo et al. 2022; Wan and Ellis 2020). These studies often highlight differences between thick-lobed cyanolichens with high STM, such as *L.scrobiculata*, and thinner-lobed chlorolichens with low STM, such as *R. farinacea* (Merinero et al. 2014). However, within the European temperate rainforest, lichen epiphyte species with morphological contrasts apparently coexist (Pentecost and Richardson 2011), and although important, morphological functional traits may have a limit to their explanatory power. In addition, we show that when morphological traits relating to water capture and retention are linked to physiological optima, they can begin to better explain how species across a spectrum of morphologies are differentially adapted or acclimated and associated with the temperate rainforest climate. This ecophysiological knowledge will become increasingly important for an accurate understanding of climate change impacts on temperate rainforest biodiversity, manifest through species biological response at a microhabitat scale driving broader distribution patterns.

## Supplementary Information

Below is the link to the electronic supplementary material.

Supplementary file1 (PDF 137 KB)

Supplementary file2 (PDF 174 KB)

## Data Availability

All data and code for reproduction of plots and data analysis are available on Github: https://github.com/AmarisOrmond.

## Code availability

All data and code for reproduction of plots and data analysis are available on Github https://github.com/AmarisOrmond.
