# Supplementary material for: The trade-off between photosynthetic rate and thallus moisture-demand explains lichen habitat association with the temperate rainforest
Source: Oecologia. 2025 Mar 5;207(3):48. doi: 10.1007/s00442-025-05687-3 (PMC11882653; doi:10.1007/s00442-025-05687-3)
Supplement: Supplementary file 1 — Supplementary file1 (PDF 137 KB) [file 442_2025_5687_MOESM1_ESM.pdf]

The trade-off between photosynthetic rate and thallus moisture-demand explains lichen habitat association with the temperate rainforest

Amaris Ormond1; Christopher J. Ellis2, Claudia Colesie1*

1 School of Geosciences, Global Change Institute, University of Edinburgh, Edinburgh, UK

2 Royal Botanic Garden Edinburgh, 20A Inverleith Row, Edinburgh, EH3 5LR, UK

*Author of correspondence: Claudia.Colesie.ed.ac.uk

Oecologia

Supplementary table 1: Mass and area measurements of seven species of lichen (*n* = *3*) (*R. virens* n = 2). Species are ordered based on increasing association with the temperate rainforest, from weakly (top) to strongly associated. Wet mass (WM) measured at the start of a series of light response curves and Dry Mass (DM) measured at the end of measurements. Thallus area measured in cm^2 using ImageJ (Fiji) 2.5

**Sample**	**Wet Mass** (WM) at start of cycle (mg)	**Dry Mass** (mg)	**Area (cm^2)**
Lobaria pulmonaria 1	736.4	210.5	20.0911
Lobaria pulmonaria 2	567	152.1	12.942
Lobaria pulmonaria 3	797.8	206.1	17.31
Ramalina calicaris 1	814.6	273.9	17.731
Ramalina calicaris 2	987	282.1	15.773
Ramalina calicaris 3	723.4	259.2	14.079
Sticta limbata 1	656.4	141.5	18.174
Sticta limbata 2	378.6	59.8	7.568
Sticta limbata 3	215.5	34.5	4.271
Sticta sylvatica 1	664.9	108.5	8.561
Sticta sylvatica 2	612.5	107.6	7.358
Sticta sylvatica 3	468.7	98.1	6.856
Ricasolia virens 2	631.1	142.1	10.146
Ricasolia virens 3	392.6	102.2	5.142
Hypotrachyna laevigata 1	769.2	144.9	10.636
Hypotrachyna laevigata 2	938.3	230.8	12.178
Hypotrachyna laevigata 3	570.5	136.2	8.114
Pectenia atlantica 1	1920.5	310.7	9.567
Pectenia atlantica 2	1752.8	257.1	5.148
Pectenia atlantica 3	1268.6	149.3	5.671
