# Supplementary material for: The trade-off between photosynthetic rate and thallus moisture-demand explains lichen habitat association with the temperate rainforest
Source: Oecologia. 2025 Mar 5;207(3):48. doi: 10.1007/s00442-025-05687-3 (PMC11882653; doi:10.1007/s00442-025-05687-3)
Supplement: Supplementary file 2 — Supplementary file2 (PDF 174 KB) [file 442_2025_5687_MOESM2_ESM.pdf]

The trade-off between photosynthetic rate and thallus moisture-demand explains lichen habitat association with the temperate rainforest

Amaris Ormond1; Christopher J. Ellis2, Claudia Colesie1*

1 School of Geosciences, Global Change Institute, University of Edinburgh, Edinburgh, UK

2 Royal Botanic Garden Edinburgh, 20A Inverleith Row, Edinburgh, EH3 5LR, UK

*Author of correspondence: Claudia.Colesie.ed.ac.uk

Oecologia

Supplementary table 2: Maximum Net Photosynthesis (MaxNP) and Maximum Dark Respiration (MaxDR) calculated as a function of lichen thallus area (cm^2) determined from desiccation curves at 18°C, saturating PPFD (µmol photons m^{-2} s^{-1}) and ambient CO_2 for seven species of lichen. Data represent the means of (*n* = *3*) (*R. virens* n = 2) replicates ± standard deviation. Species are ordered based on increasing association with the temperate rainforest, from weakly (top) to strongly associated. Chlorophyll contents (Chl) were calculated per thallus area and per Dry Mass (DM) using methods described in (Arnon and McSwain, 1974) and presented as Ch-la +b and Chl-a only. MaxNP, MaxDR, Chl-a+b area, Chl-a area and Chl-a DM showed no significant differences between species: (F =1.44, *p* = .27), (F = 2.08 , *p* = .13), (F = 1.46, *p* = .28), (F = 2.33 , *p* = 009) and (F = 2.73, *p* = .06), respectively. Values with the same small letter are not significantly different.

**Species**	**MaxNP** (nmol m^{-2} s^{-1})	**MaxDR** (nmol $g^{-2}m^2$ s^{-1})	**Chl-a+b (area based)** (ug Chl-a+b / mg m^{-2})	**Chl-a (area based)** (ug Chl-a / mg m^{-2})	**Chl-a (dry mass based)** (ug Chl-a / mg DW^{-2})
Lobaria pulmonaria	241.58 ± 82.8^a	-47.3 ± 14.4^a	1.1 ± 0.8^a	21.3 ± 19.9^a	5.1 ± 0.5^a
Ramalina calicaris	206.02 ± 31.9^a	-34.9 ± 8.4^a	0.7 ± 0.4^a	10.6 ± 7.4^a	6.2 ± 2.0^a
Sticta limbata	310.75 ± 132.4^a	-51.8 ± 17.9^a	0.4 ± 0.2^a	13.4 ± 1.3^a	5.9 ± 1.3^a
Sticta sylvatica	206.74 ± 46.2^a	-50.2 ± 9.8^a	0.9 ± 0.2^a	19.2 ± 3.1^a	4.1 ± 1.1^a
Ricasolia virens	163.05 ± 22.3^a	-86.1 ± 32.6^a	1.3 ± 0.7^a	46.2 ± 31.5^a	2.0 ± 0.5^a
Hypotrachyna laevigata	156.98 ± 58.1^a	-48.2 ± 27.8^a	0.5 ± 0.1^a	7.4 ± 2.5^a	3.9 ± 1.8^a
Pectenia atlantica	252.77± 74.4^a	-78.2 ± 26.4^a	0.9 ± 0.2^a	17.6 ± 7.4^a	3.3 ± 0.2^a
